# Access to medicines: relations with the institutionalization of pharmaceutical services

**DOI:** 10.11606/S1518-8787.2017051007138

**Published:** 2017-09-22

**Authors:** Rafael Damasceno de Barros, Ediná Alves Costa, Djanilson Barbosa dos Santos, Gisélia Santana Souza, Juliana Álvares, Augusto Afonso Guerra, Francisco de Assis Acurcio, Ione Aquemi Guibu, Karen Sarmento Costa, Margô Gomes de Oliveira Karnikowski, Orlando Mario Soeiro, Silvana Nair Leite

**Affiliations:** I Instituto de Saúde Coletiva. Universidade Federal da Bahia. Salvador, BA, Brasil; II Centro de Ciências da Saúde. Universidade Federal do Recôncavo da Bahia. Santo Antônio de Jesus, BA, Brasil; III Faculdade de Farmácia. Universidade Federal da Bahia. Salvador, BA, Brasil; IV Departamento de Farmácia Social. Faculdade de Farmácia. Universidade Federal de Minas Gerais. Belo Horizonte, MG, Brasil; V Departamento de Saúde Coletiva. Faculdade de Ciências Médicas. Santa Casa de São Paulo. São Paulo, SP, Brasil; VI Núcleo de Estudos de Políticas Públicas. Universidade Estadual de Campinas. Campinas, SP, Brasil; VII Programa de Pós-Graduação em Saúde Coletiva. Departamento de Saúde Coletiva. Faculdade de Medicina. Universidade Estadual de Campinas. Campinas, SP, Brasil; VIII Programa de Pós-Graduação em Epidemiologia. Faculdade de Medicina. Universidade Federal do Rio Grande do Sul. Porto Alegre, RS, Brasil; IX Faculdade de Ceilândia. Universidade de Brasília. Brasília, DF, Brasil; X Faculdade de Ciências Farmacêuticas. Pontifícia Universidade Católica de Campinas. Campinas, SP, Brasil; XI Departamento de Ciências Farmacêuticas. Universidade Federal de Santa Catarina. Florianópolis, SC, Brasil

**Keywords:** Pharmaceutical Services, organization & administration, Health Services Accessibility, Primary Health Care, Health Services Research, Unified Health System, Assistência Farmacêutica, organização & administração, Acesso aos Serviços de Saúde, Atenção Primária à Saúde, Pesquisa sobre Serviços de Saúde, Sistema Único de Saúde

## Abstract

**OBJETIVE:**

To analyze the relationship between access to medicines by the population and the institutionalization of pharmaceutical services in Brazilian primary health care.

**METHODS:**

This study is part of the *Pesquisa Nacional sobre Acesso, Utilização e Promoção do Uso Racional de Medicamentos – Serviços 2015* (PNAUM – National Survey on Access, Use and Promotion of Rational Use of Medicines – Services 2015), a cross-sectional, exploratory, and evaluative study composed of an information survey in a representative sample of cities, stratified by Brazilian regions. Access was defined based on the acquisition of medicines reported by the patient, ranging between: total, partial, or null. The institutionalization of pharmaceutical services was analyzed based on information provided by pharmaceutical services providers and by those responsible for medicines delivery. Chi-square test and multinomial logistic regression were used in the statistical analysis.

**RESULTS:**

Full access to medicines was greater when professionals affirmed there were the following aspects of the dimensions: “management tools,” “participation and social control,” “financing,” and “personnel structure,” with significant associations in the bivariate analysis. The “pharmaceutical care” dimension did not achieve such an association. After multinomial logistic regression, full access was more prevalent when those in charge of pharmaceutical services stated that: they always or repeatedly attend meetings of the Municipal Health Council, OR = 3.3 (95%CI 1.5-7.3); there are protocols for medicines delivery, OR = 2.7 (95%CI 1.2-6.1); there is computerized system for managing pharmaceutical services, OR = 3.9 (95%CI 1.9-8.0); those responsible for medicines delivery reported having participated in a course or training for professionals in the past two years, OR = 2.0 (95%CI 1.1-3.5); there is computerized system for pharmaceutical services management, OR = 4.3 (95%CI 2.4-7.5).

**CONCLUSIONS:**

Aspects related to the institutionalization of pharmaceutical services have been strongly related to access to medicines. Our results indicate the need to prioritize its implementation, contributing to its consolidation in Brazil and to the effectiveness of health services regarding the purposes of pharmaceutical services policies.

## RESUMO

**OBJETIVO:**

Analisar as relações entre o acesso a medicamentos pela população e a institucionalização da assistência farmacêutica, na atenção básica.

**MÉTODOS:**

Este artigo integra a Pesquisa Nacional sobre Acesso, Utilização e Promoção do Uso Racional de Medicamentos – Serviços 2015, estudo transversal, exploratório, de natureza avaliativa, composto por um levantamento de informações em amostra representativa de municípios, estratificada pelas regiões do Brasil. O acesso a medicamentos foi definido a partir do referencial de obtenção pelo usuário, variando entre: total, parcial ou nulo. A institucionalização da assistência farmacêutica foi analisada a partir das informações prestadas por responsáveis pela assistência farmacêutica e por responsáveis pela entrega de medicamentos. Na análise estatística foram utilizados o teste Qui-quadrado e regressão logística multinomial.

**RESULTADOS:**

O acesso total a medicamentos foi maior quando os profissionais afirmaram haver aspectos das dimensões: “ferramentas de gestão”, “participação e controle social”, “financiamento” e “estrutura de pessoal”, constatando-se associações significantes na análise bivariada. A dimensão “atenção farmacêutica” não obteve tal associação. Após regressão logística multinomial, o acesso total foi mais prevalente quando responsáveis pela assistência farmacêutica afirmaram: participar sempre ou repetidamente das reuniões do Conselho Municipal de Saúde, RP= 3,3 (IC95% 1,5–7,3); existir protocolos para a entrega de medicamentos, RP= 2,7 (IC95% 1,2–6,1); existir sistema informatizado para gestão da assistência farmacêutica, RP= 3,9 (IC95% 1,9–8,0); e quando responsáveis pela entrega de medicamentos afirmaram ter participado de curso ou capacitação para profissionais nos últimos dois anos, RP= 2,0 (IC95% 1,1–3,5) e haver sistema informatizado para gestão da assistência farmacêutica, RP= 4,3 (IC95% 2,4–7,5).

**CONCLUSÕES:**

Aspectos relacionados com a institucionalização da assistência farmacêutica se mostraram fortemente relacionados com o acesso a medicamentos. Os resultados indicam a necessidade de priorizar a sua implementação, contribuindo para sua consolidação no Brasil e efetividade dos serviços de saúde quanto às finalidades das políticas de assistência farmacêutica.

## INTRODUCTION

The medicine is considered a social asset and something essential in the functioning of health services^4^, yet its access in the world is still irregular. Data from the World Health Organization^24^ show that one-third of the world’s population still does not have regular access to this technology. The explanations for this fact may be the lack of universal social protection systems in health and the low purchasing power of governments and populations of these countries, besides the high rates of waste in pharmaceutical services^4^.

Brazil is not outside this context. Since the creation of the Brazilian Unified Health System (SUS), the country has undergone an intense process of organization of its health system, in which medicines are regarded as essential tools in the health care process^14^.

Despite the recognition of improvements in health care since the creation of SUS^31^, a specific national policy for medicines and pharmaceutical services^18^ (PS) was only established in 1998.

The main purposes of the *Política Nacional de Medicamentos* (PNM – National Drug Policy) are: ensuring the safety, efficacy, and quality of medicines; promoting the rational use and access of the population to essential medicines. To achieve the defined objectives, it established guidelines, including the reorientation of pharmaceutical services. In 2004, the *Política Nacional de Assistência Farmacêutica* (PNAF – National Policy of Pharmaceutical Services)^19^, which provides for its reorientation, deepening the principles and strategic axes of action, was approved.

Since the 1990s, SUS began a strong process of expansion of health services^21^. In view of this expansion, there was also a need for reorganization of PS in the SUS, to increase the coverage of free distribution of medicines and at the same time minimize costs.

Access to medicines integrates the access to health services and care^25^. Therefore, the organization of PS is part of the health system.

The process of institutionalizing a public policy presupposes that social behaviors, obligations, or realities have to assume a status of rule in social thought and action^17^. Thus, the institutionalization of PS, based on its reorientation process defined by the PNM, may have been determinant in the access to medicines by the Brazilian population in the past years, mainly in primary health care.

This study aimed to analyze, in a multidimensional perspective, the access to medicines by the population and the institutionalization of PS in SUS primary health care. It presents an approach to the institutionalization of PS not yet found in publications on access to medicines and sociodemographic factors^15^ ^,^ ^16^ ^,^ ^20^ ^,^ ^22^.

## METHODS

This study’s data are part of the *Pesquisa Nacional sobre Acesso, Utilização e Promoção do Uso Racional de Medicamentos – Serviços 2015* (PNAUM – National Survey on Access, Use and Promotion of Rational Use of Medicines – Services 2015), which aimed to characterize the organization of PS in the primary health care of SUS – for promoting the access and rational use of medicines –, as well as to identify and discuss the factors that interfere in the consolidation of PS in the cities.

PNAUM – Services is the most comprehensive research on PS in Brazil. It is an exploratory, evaluative study, consisting of an information survey in a sample of primary health care services in 600 cities that represent the Brazilian regions, with direct observation of 1,175 pharmaceutical services and face-to-face and remote interviews. Several study populations were considered in the sampling plan, with samples stratified by the regions, which are domains of the study. Interviews were conducted by trained interviewers, using a specific questionnaire for each category of interviewee; performed in person with those responsible for the delivery of medicines (RDM), (n = 1,139 or 83.6% of the estimated sample) and with users (n = 8,803 or 97.8% of the estimated sample), with collection and storage of data in an electronic device (tablet computer). Remotely, by a telephone interview, with those responsible for pharmaceutical services (RPS) (n = 507 or 84.5% of the estimated sample), with data storage also in electronic device (tablet computer). Details on the sampling and logistics of data collection are described in the methodological article of PNAUM – Services^1^.

As in other PNAUM studies^2^ ^,^ ^17^ ^,^ ^20^, access to medicines was analyzed according to the users’ information. We used the question: “In the past three months, how often did you get the medicines you were looking for in the Public Pharmacies of SUS?.” Access was classified as full, partial, and null. We considered full access when users always obtained the needed medicines in the past three months; partial, when they obtained them repeatedly, sometimes, or rarely; and null, when they never obtained the sought medicines in the past three months.

The institutionalization of PS was analyzed from the results of the interviews with RPS and RDM, according to the assumption that these actors are the most involved with PS in the aspects of their institutionalization.

For the purposes of this article, the user sample was redefined:

A - From the total number of users interviewed in PNAUM (n = 8,803), we considered only those who answered affirmatively to the question “In the past three months, did you look for medicines in SUS public pharmacies?.” The database of those who answered affirmatively (n = 5,758) was merged with RPS and RDM databases, generating two new study populations: one related to aspects reported by RPS and other to those reported by RDM.

B1 - In the process of merging user and RPS databases, we only included users who claimed to live in cities where the RPS was also interviewed, resulting in the first study population (n = 4,866). The key variable, mandatorily present and equal in these databases, was the city declared by both RPS and user.

B2 - In the merger of the user and RDM databases, we only included users who reported having looked for medicines at health facilities where the RDM was also interviewed, generating the second study population (n = 4,424). The key variable was the number of the health unit in the National Register of Health Establishments, present in the databases of both interviewees.

We adopted an operational concept of a study of PNAUM^27^, in which the institutionalization of PS is defined as a social and political-administrative process. This process is expressed in the creation and implementation of formal structures in the health system, in the organization of services, in structure and mechanisms of financing, management tools, involvement in social participation and control, and conduction of practices and activities inherent to PS as a component of comprehensive health care. Based on this operational concept, we defined the following dimensions of the institutionalization process of PS for this study: formal structures of PS in the *Sistema de Saúde Municipal* (SSM – Municipal Health System); personnel structure; financing; management tools; participation and social control; pharmaceutical care.

The dimensions of “formal structures of PS in the SSM,” “financing,” and “participation and social control” were analyzed with information provided by those RPS. “Personnel structure” and “pharmaceutical care,” with information by those RDM. “Management tools,” based on the two questionnaires.

The “pharmaceutical care” dimension was analyzed from the variable “performance of clinical activity,” legally exclusive to the pharmaceutical professional^7^; therefore, we only included users who claimed to seek medicines at health units where the RDM was a pharmacist.

The merging of databases and the data analysis were performed using SPSS® software, version 21. In the bivariate analysis, Pearson’s Chi-square test was used to verify the association between access to medicines (full, partial, and null) and aspects of the institutionalization of PS. In the multivariate analysis, a multinomial logistic regression was performed^10^ to estimate the association between access to medicines and variables related to the institutionalization of PS, since the response variable has three categories. The model results were presented as odds ratios (OR) and with 95% confidence intervals. The criterion for the inclusion of variables in the multinomial logistic model was the association of variables with access at p < 0.20 level in the bivariate analysis. For the permanence of the variables in the multinomial logistic model, the level of p < 0.05 was used.

PNAUM – Services was approved by the National Research Ethics Committee (Opinion 398.131/2013). The objectives of the research were explained to the interviewees, and they signed the informed consent form.

## RESULTS

Table 1 presents the results of access to medicines by users, according to aspects of institutionalization, informed by those RPS. In the “formal structures of PS in the SSM” dimension, the existence of exclusive *Comissão Permanente de Licitação* (CPL – Permanent Bidding Committee) was the only variable with significant association with access to medicines: full access (64.0%) was 5.9% higher than in places without such commission (58.1%). The variables “presence of PS in the Municipal Health Plan,” “existence of Standardized List of Medicines,” and “Pharmacy and Therapeutics Committee” did not obtain significant association.

**Table 1:** Aspects of access to medicines according to information provided by those responsible for pharmaceutical services. National Survey on Access, Use and Promotion of Rational Use of Medicines – Services, 2015.

Dimension	Variable	Users (n)	Access % (95CI%)			p*

			Full	Partial	Null	
Formal PS structures in the SSM	Pharmaceutical services are included in the Municipal Health Plan					0.324
	Yes	4,361	60.6 (58.6–62.6)	35.3 (33.3–37.3)	4.1 (3.3–5.0)	
	No	268	54.2 (46.8–61.4)	43.2 (36.1–50.6)	2.6 (0.9–6.9)	
	Existence of Pharmacy and Therapeutics Committee					0.102
	Yes	2,268	57.5 (54.3–60.6)	39.4 (36.3–42.6)	3.1 (2.2–4.4)	
	In the implementation phase	483	66.1 (60.2–71.6)	30.5 (25.3–36.2)	3.4 (1.7–6.7)	
	No	1,993	61.0 (58.4–63.5)	34.6 (32.2–37.1)	4.5 (3.5–5.7)	
	Existence of Standardized Medicines List					0.878
	Yes	4,468	60.1 (58.1–62.1)	35.7 (33.8–37.7)	4.1 (3.4–5.1)	
	No	316	61.8 (55.1–68.1)	34.6 (28.5–41.1)	3.7 (1.8–7.5)	
	Existence of an exclusive Permanent Commission for the purchase of medicines					0.012
	Yes	1,666	64.0 (61.0–67.0)	32.7 (29.8–35.7)	3.3 (2.4–4.6)	
	No	3,042	58.1 (55.6–60.6)	37.7 (35.2–40.2)	4.2 (3.3–5.4)	
Financing	Expenses with the structuring of PS					0.417
	Yes	2,484	59.2 (56.4–61.9)	36.8 (34.2–39.6)	3.9 (3.0–5.2)	
	No	1,969	61.5 (58.6–64.3)	35.3 (32.5–38.2)	3.2 (2.3–4.4)	
	Coordination of PS has autonomy in the management of financial resources					0.004
	Yes, completely	924	60.8 (56.9–64.5)	36.5 (32.9–40.3)	2.7 (1.7–4.2)	
	Yes, partially	2,324	60.8 (57.9–63.6)	35.3 (32.6–38.2)	3.9 (2.9–5.3)	
	No	1,445	56.9 (53.3–60.4)	36.9 (33.5–40.5)	6.2 (4.6–8.3)	
Management tools	Existence of computerized system for PS management					< 0.001
	Yes	3,722	62.7 (60.5–64.8)	34.2 (32.2–36.4)	3.1 (2.4–3.9)	
	No	1,093	52.8 (48.7–56.8)	40.3 (36.4–44.4)	6.9 (5.0–9.3)	
	Existence of protocol for the storage of medicines					< 0.001
	Yes	3,449	64.3 (62.0–66.5)	32.6 (30.4–34.9)	3.1 (2.4–4.0)	
	No	1,202	51.1 (47.5–54.8)	43.1 (39.5–46.8)	5.8 (4.2–7.9)	
	Existence of protocol for the dispensing of medicines					< 0.001
	Yes	3,268	64.8 (62.4–67.1)	32.0 (29.7–34.3)	3.2 (2.5–4.2)	
	No	1,418	52.3 (49.0–55.5)	42.4 (39.2–45.4)	5.3 (4.0–7.1)	
	Existence of protocol for the delivery of medicines					< 0.001
	Yes	3,027	61.9 (59.5–64.3)	35.0 (32.7–37.4)	3.1 (2.4–4.0)	
	No	1,591	54.6 (51.1–58.0)	39.1 (35.8–42.5)	6.3 (4.7–8.4)	
	Existence of control of entry and exit of medicines from the warehouse					0.951
	Yes	4,625	60.5 (58.5–62.4)	35.6 (33.7–37.5)	3.9 (3.2–4.8)	
	No	190	60.3 (51.7–68.3)	36.2 (28.5–44–8)	3.4 (1.3–8.4)	
	Existence of some type of training or qualification of the PS professionals					< 0.001
	Yes	872	73.0 (69.0–76.7)	25.6 (22.0–29.5)	1.4 (0.8–2.7)	
	No	2,951	56.4 (54.0–58.9)	38.5 (36.1–41.0)	5.0 (4.0–6.3)	
Participation and social control	Existence of mechanisms for receiving criticism by users about PS					0.051
	Yes	1,938	62.6 (59.6–65.5)	32.3 (29.6–35.2)	5.1 (3.8–6.8)	
	No	1,971	58.3 (55.2–61.3)	38.1 (35.1–41.1)	3.6 (2.7–4.9)	
	Participation of the RPS in the Municipal Health Council					0.007
	Always or repeatedly	1,138	65.6 (62.0–69.1)	31.8 (28.4–35.3)	2.6 (1.6–4.1)	
	Sometimes or rarely	921	59.5 (55.0–63.9)	36.9 (32.7–41.4)	3.6 (2.3–5.5)	
	Never	1,493	59.2 (56.0–62.4)	35.8 (32.7–38.9)	5.0 (3.7–6.7)	

PS: pharmaceutical services; SSM: Municipal Health System; RAF: responsible for pharmaceutical services

* Significant association: p < 0.05

Source: PNAUM – Services, 2015.

In the “financing” dimension, the results of full access to medicines regarding the spending on the structuring of PS were close between places where the RPS stated that there was no expenditure (61.5%) and places where there was expenditure (59.2%). There was no significant association between the two variables.

Regarding the variable “PS Coordination autonomy in the management of financial resources,” full access to medicines was equal when the autonomy was full or partial (60.8%) and lower when there was no autonomy (56.9%), with significant association.

In the “management tools” dimension, full access to medicines was greater when the RPS stated that: there was computerized system for PS management; protocols for storage, dispensing, and delivery of medicines; some type of qualification or training for the PS professionals. These variables obtained significant association.

In the “participation and social control” dimension, full access to medicines was greater where there were mechanisms to receive criticism about the PS by users, without significant association. Regarding the participation of those RPS in the *Conselho Municipal de Saúde* (CMS – Municipal Health Council), full access (65.6%) was higher when professionals affirmed they always or repeatedly participated in CMS, with significant association.

Table 2 presents the results of access to medicines by users, according to aspects of institutionalization reported by those RDM. In the “personnel structure” dimension, 29% of users sought medicines at health facilities where the RDM was a pharmacist, 8.4% with a pharmacy technician or assistant, 10.7% with a nurse, 29.7% with a nursing assistant or technician, 8.7% with administrative assistant, and 2.5% with community health agents.

**Table 2:** Aspects of access to medicines according to information provided by those responsible for the delivery of medicines. National Survey on Access, Use and Promotion of Rational Use of Medicines – Services, 2015.

Dimension	Variable	Users (n)	Access % (95%CI)			p*

			Full	Partial	Null	
Personnel structure	Professional category of the RDM					< 0.001
	Pharmacist	1,282	64.9 (61.5–68.3)	32.3 (29.0–35.6)	2.8 (1.9–4.2)	
	Pharmacy assist./tech.	370	67.4 (60.8–73.4)	30.3 (24.5–36.8)	2.3 (1.0–5.1)	
	Nurse	474	56.6 (51.0–62.1)	38.9 (33.6–44.5)	4.5 (2.6–7.5)	
	Nursing assist /tech.	1,312	61.2 (57.2–64.9)	36.0 (32.3–39.9)	2.8 (1.8–4.3)	
	Administrative assist.	387	50.4 (42.5–58.3)	35.8 (28.7–43.5)	13.8 (8.7–21.0)	
	Community health agent	112	43.9 (29.9–58.9)	53.2 (38.3–67.6)	2.9 (0.6–13.1)	
	Others	487	58.2 (51.9–64.3)	35.2 (29.5–41.4)	6.6 (3.4–5.0)	
Management tools	Participation in training or course for PS professionals in the city in the past two years					0.009
	Yes	1,491	65.3 (61.8–68.6)	30.3 (27.1–33.6)	4.5 (3.1–6.4)	
	No	2,922	59.1 (56.7–61.6)	36.9 (34.5–39.3)	4.0 (3.1–5.0)	
	Existence of computerized system for PS management					< 0.001
	Yes	1,979	65.7 (62.8–68.4)	32.0 (29.3–34.8)	2.4 (1.6–3.4)	
	No	2,399	57.8 (55.0–60.6)	36.8 (34.1–39.5)	5.4 (4.3–6.8)	
	Existence of a computerized system for PS management networked with health units					< 0.001
	Yes	1,533	70.3 (67.1–73.2)	28.0 (25.1–31.1)	1.7 (1.0–2.8)	
	No	418	46.3 (39.9–52.9)	48.2 (41.7–54.7)	5.5 (3.0–9.8)	
	Pharmacist participates of the scheduling of medicines in the health unit					0.015
	Yes	2,931	62.8 (60.3–65.3)	32.6 (30.3–35.1)	4.6 (3.6–5.8)	
	No	1,387	58.3 (54.9–61.7)	38.3 (34.9–41.7)	3.4 (2.4–4.8)	
	Pharmacist participates in the stock control in the health unit					< 0.001
	Yes	3,377	64.2 (61.9–66.5)	32.0 (29.8–34.3)	3.8 (3.0–4.8)	
	No	1,018	52.1 (48.2–56.1)	42.8 (38.9–46.7)	5.1 (3.5–7.3)	
	Pharmacist participates of the medicines dispensing/delivery in the health unit					< 0.001
	Yes	3,045	63.9 (61.5–66.2)	32.1 (29.9–34.5)	4.0 (3.1–5.1)	
	No	1,373	54.7 (51.1–58.2)	40.8 (37.3–44.4)	4.5 (3.2–6.3)	
	Existence of an inventory control system (entry and exit) of medicines					< 0.001
	Yes, manual	2,327	57.8 (54.9–60.6)	36.9 (34.2–39.7)	5.3 (4.2–6.8)	
	Yes, computerized	1,932	65.7 (62.8–68.4)	31.8 (29.2–34.7)	2.5 (1.7–3.5)	
	No	160	60.1 (48.1–71.0)	35.5 (25.1–47.4)	4.5 (1.4–13.4)	
	Existence of control of the expiration date of medicines					< 0.001
	Yes, manual	2,902	57.1 (54.5–59.6)	37.8 (35.3–40.3)	5.1 (4.1–6.4)	
	Yes, computerized	1,437	69.7 (66.5–72.7)	28.5 (25.5–31.6)	1.8 (1.2–2.8)	
	No	80	53.4 (37.9–68.3)	36.7 (23.1–52.9)	9.9 (3.7–23.7)	
Pharmaceutical care	Performing a clinical activity					0.638
	Yes	343	67.5 (60.2–74.0)	29.3 (23.1–36.5)	3.2 (1.4–6.8)	
	No	939	64.3 (60.4–68.1)	33.0 (29.3–36.8)	2.7 (1.7–4.3)	

RDM: responsible for the delivery of medicines; PS: pharmaceutical services.

* Significant association: p ≤ 0.05

Source: PNAUM – Services, 2015.

Full access was higher when the RDM was a pharmacist (64.9%) or pharmacy assistant/technician (67.4%), decreasing when they were another professional. The lowest percentages of full access were found when the RDM was an administrative assistant (50.4%) and community health agent (43.9%). This variable showed significant association.

In the “management tools” dimension, full access to medicines was greater when there was: participation of the RDM in training or courses on PS in the past two years; computerized system for PS management; connection of the computerized network system with health units; participation of the pharmacist in the scheduling, stock control and dispensing/delivery of medicines; control system of inventory and of expiration date of medicines. All tools analyzed had a significant association with access to medicines.

Similar to the results according to the RPS, full access (65.7%) was higher when RDM stated there was a computerized system compared to places without the same technology (57.8%). Where this system was networked with health units, full access was even higher (70.3%). It was also higher when the RDM stated that the pharmacist participated in the scheduling of medicines of health units (62.8%), inventory control (64.2%), and dispensing/delivery of medicines (63.9%).

We observed that, when the control of inventory and of expiration date of medicines were computerized, the percentages of full access were higher, mainly regarding the expiration date control, which was 12.6% higher than the places where the control was manual.

In the “pharmaceutical care” dimension, full access (67.5%) was higher when the RDM claimed to perform this activity. This aspect did not show significant association.

Tables 3 and 4 present the results of the multivariate analysis, by multinomial logistic regression, of institutionalization aspects related to the RPS and RDM, respectively.

**Table 3:** Results of the multivariate analysis of aspects of the institutionalization of pharmaceutical services associated with access to medicines according to the information of those responsible for pharmaceutical services. National Survey on Access, Use and Promotion of Rational Use of Medicines – Services, 2015.

Variable	Null Access OR	Partial Access OR (95%CI)^*^		Full Access OR (95%CI)^*^	
Participation of the RPS always or repeatedly at the Municipal Health Council	1.0	2.3	(1.0–5.1)	3.3	(1.5–7.3)
Existence of protocol for the delivery of medicines	1.0	3.0	(1.3–6.8)	2.7	(1.2–6.1)
Existence of computerized system for PS management	1.0	4.0	(1.9–8.2)	3.9	(1.9–8.0)
Existence of protocol for the storage of medicines	1.0	1.1	(0.4–2.5)	2.9	(1.2–6.9)

RPS: responsible for pharmaceutical services

^*^ OR (95%CI) – Odds Ratio (95% confidence interval). For this analysis, the reference was the category of “Null Access” to medicines by users.

Source: PNAUM – Services, 2015.

**Table 4:** Results of the multivariate analysis of aspects of the institutionalization of pharmaceutical services associated with access to medicines according to the information of those responsible for the delivery of medicines. National Survey on Access, Use and Promotion of Rational Use of Medicines – Services, 2015.

Variable	Null Access OR	Partial Access OR (95%CI)^*^		Full Access OR (95%CI)^*^	
Participation in training or course for PS professionals in the city in the past two years	1.0	1.5	(0.8–2.7)	2.0	(1.1–3.5)
Existence of computerized system for PS management	1.0	2.8	(1.6–4.9)	4.3	(2.4–7.5)

PS: pharmaceutical services.

^*^ OR (95%CI) – Odds Ratio (95% confidence interval). For this analysis, the reference was the category of “Null Access” to medicines by users.

Source: PNAUM – Services, 2015.

In the multivariate analysis, some variables lost the significant association, one remaining related to the “participation and social control” dimension and three related to “management tools,” which had significant association with full access to medicines (Table 3).

Full access to medicines was more prevalent when the RPS claimed participating always or repeatedly of CMS meetings (OR = 3.3, 95%CI 1.5-7.3). There was also a higher prevalence of full access when the RPS reported that there were protocols for delivery (OR = 2.7, 95%CI 1.2-6.1) and medicine storage (OR = 2.9, 95%CI 1.2- 6.9). The highest prevalence of full access occurred when the RPS said there was computerized system for PS management (OR = 3.9, 95%CI 1.9-8.0).

Full access to medicines was more prevalent when the RDM reported having participated in a course or training for PS professionals in the past two years (OR = 2.0, 95%CI 1.1-3.5), and that there was a computerized system for PS management (OR = 4.3, 95%CI 2.4-7.5), with significant association in the multivariate analysis (Table 4).

## DISCUSSION

When analyzing the association between access to medicines and formal structures of PS in the SSM, greater access to medicines was found when the RPS claimed there was exclusive CPL for medicines acquisition. The significant association reinforces the relevance of this structure to the organization and scope of PS purposes. The lack of medicines can seriously compromise health services, and one must consider this technology a differential input during the bidding process^3^ ^,^ ^26^. The *Comissão de Farmácia e Terapêutica* (PTC – Pharmacy and Therapeutic Commitee) is another strategic framework^11^ ^,^ ^13^ for PS, as well as the *Relação Nacional de Medicamentos Essenciais* (Rename – National List of Essential Medicines)^32^.

The PNM establishes the adoption of Rename as a guideline for the strengthening of PS. According to the PNAF, the use of these lists is a strategic axis, rationalizing actions in the various areas of PS. The concept of essential medicines is widely accepted as a powerful means of promoting health equity, and it is instrumented by a standardized reference list^6^. In this study, no significant association was found between access to medicines and PTC, and standardized lists. We signal as a limitation the fact that PNAUM – Services was not able to analyze the way these structures operate and operationalize, which can affect the access to medicines.

Regarding financing, access to medicines and spending on the structuring of PS did not show a significant association. The structuring of PS is a late and unfinished process in SUS, and it covers several aspects^30^. In this sense, the question used in PNAUM (”Has the city spent on the structuring of PS in the past year?”) does not explain which aspects of this structure would be considered, allowing different interpretations of the RPS on this subject. More precise definitions and appropriate methodologies would be needed to analyze this issue. The autonomy of the RPS in the management of financial resources showed significant association. This autonomy can allow the RPS more agility in solving specific problems in PS management, which can directly interfere in the availability of medicines and consequently in their access. As Vilasbôas and Paim^32^ point out regarding the functioning of the public network, low financial autonomy affects negatively the professionalization and planning of purchases and contracts.

The variables of the “management tools” dimension expressed a strong relation with the access to medicines according to both those RPS and RDM, except for the variable “control of entry and exit of medicines in the warehouse,” in the case of those RPS. An efficient inventory control system can subsidize medicine scheduling and procurement, correcting distortions and avoiding losses^5^. Vieira^30^ found lack or deficiency in this control in 71% of the sample of studied cities^30^, and another study that evaluated PS in Brazil found records of inventory in only 32% of health units^23^. In comparison to these, PNAUM – Services showed progress in the control of entry and exit of medicines in pharmacies/public dispensing units (96%, according to the RDM).

Regarding computerized systems in PS management, 55.6% of users sought medicines in units that did not have such a system. In the units where there were, 18.5% were not connected in a network with other units, which may hinder the process of articulating the information. A study carried out in 2000 in the state of São Paulo showed that the decentralized model of distribution and dispensing of medicines, with a good information system, is more efficient and economical^8^.

In the multivariate analysis, the existence of a computerized system for PS management maintained a significant association in both study populations (RPS and RDM), presenting the highest odds ratios of full access. A study showed that the cities that adopted the model Hórus, made available by the Brazilian Ministry of Health, had an increase in the access to medicines by the population. The authors emphasize that the computerized system contributes with greater safety in the access and use of medicines, by strengthening the medicines control and monitoring process^9^.

The protocols (storage, dispensing, and delivery of medicines), are directly related to the actions of the logistics cycle of PS. The positive results of full access in relation to these instruments reinforce the hypothesis that the use of protocols is one of the strategies that have contributed to direct the rational use of medicines^29^. Two of these protocols – medicine storage and delivery – remained in with significant association the multivariate analysis; full access to medicines was more prevalent where RPS claimed these protocols exist.

The training or qualification of PS professionals in primary health care remained with significant association after the logistic regression in the multivariate analysis, with full access being more prevalent when the RDM and RPS stated that these activities occurred. The qualification of professionals is a relevant aspect in the consolidation of public policies^31^. The results of this study corroborate this statement.

In the “participation and social control” dimension, the participation of the RPS in the Municipal Health Council, informed as always or repeatedly, remained with significant association in the multivariate analysis. This finding reinforces the importance of municipal health councils as spaces for monitoring the implementation of health policies^28^.

In the “pharmaceutical care” dimension, there was no significant association between access to medicines and performance of clinical activity. It involves macro components, such as health education, pharmaceutical orientation, dispensing, pharmaceutical care, and drug therapy follow-up, as well as systematic recording of activities, measurement and evaluation of results^12^. It is necessary to indicate that, in the PNAUM studies, the access to medicines was considered from the time of their acquisition, but other dimensions of access are also considered relevant^22^ ^,^ ^25^. These other macro components were not investigated in this study, since the question used was: “Do you perform any activity that has a clinical character?” Thus, pharmaceutical care may be related to these other macro components, which would require more in-depth studies.

This study, which integrates PNAUM – Services, is a “photograph” of a specific moment of the PS organization in Brazilian primary health care, which is still in the process of implementing its policy. In view of the scarcity of other national surveys that deal with the same topic, it is considered a limitation not to have parameters for comparison of results. Other studies are needed and recommended.

However, we could identify aspects related to the institutionalization of PS that were strongly related to access to medicines. Our results indicate the need to prioritize the implementation of these aspects so that there is a consolidation of PS in Brazil, seeking the effectiveness of health services regarding the purposes of PS policies.
